# Insecticidal Activity of Grape Pomaces from Two Grape Cultivars Against the Housefly (*Musca domestica* L*.)* Under Laboratory Conditions

**DOI:** 10.21315/tlsr2018.29.2.7

**Published:** 2018-07-06

**Authors:** Abdul-Lateef Molan, Mohamad Q. Balasim, Nagham Y. Al-Bayati

**Affiliations:** 1 Department of Biotechnology, College of Sciences, Diyala University, Diyala, Iraq; 2 Department of Biology, College of Education for Pure Sciences, Diyala University, Diyala, Iraq

**Keywords:** Botanical Insecticide, Housefly, Grape Pomace, Seeds, Peels

## Abstract

Aqueous extracts prepared from the powdered entire pomaces and their parts (seeds and peels) of two Iraqi grape cultivars (Shada and Des-Alanez) were evaluated for their ovicidal, larvicidal, and pupicidal activities against housefly, *Musca domestica* (Linnaeus 1758) and compared them with chemical insecticide (Agita 10 WG; Austria). The highest insecticidal activity was shown by the aqueous extracts of powdered seeds followed by the entire pomace and then peels. The water extracts from the seeds, peels and pomaces were able to inhibit completely the hatching of the eggs at a concentration of 150 μg/ml while the Agita insecticide was able to inhibit the hatching process at a concentration of 50 μg/ml. The water extracts also were able to kill the L_3_ larvae and the activity was increasing with the increasing of the concentration. It is very interesting to note that the addition of the powdered entire pomaces, seeds, and peels on top or at the bottom of the cow manure was able to inhibit the development of up to 50% and 47.5% of L_1_ to pupae and to adult flies, respectively. Taking into consideration the huge amounts of grape pomaces that produced annually, the cheap price and the ease in dealing with, make them a good candidate as alternative insecticides and environmentally more friendly than the chemical insecticides.

## INTRODUCTION

Globally, grape reaches the 16th rank of the most harvested food and agriculture commodities with almost 67 million metric tonnes (Food and Agriculture Organization [FAO] 2009). In New Zealand, for example, 328,000 tons of grapes have been crushed in 2011 to produce 325,000 million litres of wine (Lee-Jones 2012). This produces 42.3 million kg of pomace (solid press remains of grapes after pressing for juice or wine and consist of skin/peel, stems, and seeds) considering 18 kg pomace/100 L wine (Rockenbach *et al*. 2011). It is well known that grape pomaces have high concentrations of macronutrients, high concentrations of polyphenols and low concentrations of micronutrients, as well as heavy metals contents which make the residues incompatible with agricultural requirements (Kammerer *et al*. 2004; Bustamante *et al*. 2008) and therefore the waste must be conditioned prior to use because they pose a significant disposal problem (Devesa-Rey *et al*. 2011).

The disadvantage of having high levels of tannins, grape pomaces and other agricultural wastes may be turned to an advantage via extraction and isolation of polyphenols before using them as animal feed or other uses. However, some tannins (especially condensed tannins) can be used positively as they have been used as anthelmintic (Molan, Alexander *et al.* 2000; Molan, Duncan *et al.* 2000; Molan, Hoskin *et al*. 2000; Molan, Waghorn *et al.* 2000; Molan *et al.* 2002; Molan & Farag 2010; Molan 2014) and antimicrobial agents (Molan *et al*. 2001).

The housefly, *Musca domestica* (Diptera: Muscidae), belongs to the Order Diptera which presents the biggest order within the Phylum Arthropoda and its members are vectors of a wide range of diseases and therefore they pose the greatest challenge to human and veterinary health (Malik *et al*. 2007; Palacios *et al.* 2009). In addition to its role in the mechanical transmission of infectious agents, housefly causes a nuisance, especially the adult stage (Khan *et al*. 2013). In addition, current method of housefly control with chemical insecticides has resulted in development of resistance (Scott *et al*. 2013), which warrant search for alternative insecticides such as agricultural wastes which are not used wisely and the grape pomace is a good example.

Although the biological activities of the pomaces of various cultivars of grapes have been studied (Lurton 2003, Ozkan *et al*. 2004, Morre & Morre 2005, Curko *et al*. 2014, Zhu *et al*. 2015), to the best of our knowledge, no previous studies have investigated the insecticidal activity of grape pomaces and/or their ingredients (peels and seeds) against the housefly, *Musca domestica*. Thus, the current study was undertaken to address this gap by evaluating the insecticidal activity of the entire pomaces and their ingredients of two Iraqi grape cultivars against the eggs, larvae and pupae of the housefly.

## MATERIALS AND METHODS

### Grape Varieties

Grape samples were obtained from local markets in Iraq. The grape varieties were Des-Alanez (white/green grape cultivar) and Shada (purple-black grape cultivar) grown in Diyala Province, middle of Iraq. All the pomace extracts were prepared from a single production lot.

### Extraction of Grape Pomaces

Samples of grape (*Vitis vinifera* L.) of the varieties Shada and Des-Alanez were purchased from the local markets of Diyala Province, Iraq (2014/2015 harvest season) they were stored under refrigeration for 48 h at 4°C until analysis. The samples were then cleaned and de-stemmed and the green unripe and old shriveled grapes were discarded. The grapes were mashed using a domestic blender and then the mixture was strained through double layers of cheesecloth. After the extraction of grape juice, the pomace was stored in polyethylene bags at −20°C until processing. Only the pomaces were used in this study, which have been dried in oven at 50°C for 72 h. The dried pomaces were divided into three lots, the entire pomace (as is), seeds, and peels. The seeds and peels were separated manually from the dried pomaces. The dried pomaces, seeds and peels were ground using domestic coffee grinder. The powdered pomaces, seeds, and peels were stored in polyethylene bags at −20°C until being used.

The powdered pomaces, seeds, and peels were used as they are or extracted in boiling distilled water as described previously (Molan & Mahdy 2016; Molan *et al.* 2017). Briefly, 400 mg of the powdered samples were mixed with 40 mL of boiling distilled water in a 50 mL-centrifuge tube, vortexed for 10 min and then left overnight. All the extracts were prepared one day prior to the commencement of the experiments, centrifuged on the second day and used immediately in the experiments.

### Rearing of Housefly

Adult house flies were collected from the garbage sites of the main campus of Diyala University, Iraq, using a sweep net method and reared as described by Kumar *et al*. (2012). The captured flies were reared in transparent cages (30 cm × 30 cm × 30 cm) with mesh screens on opposite sides and a cloth sleeve opening at the front (Farooq & Freed 2016) in the insect rearing room of our laboratory at 25°C–28°C, and 55%–60% relative humidity and fed a sugar/ milk solution (50 g of powdered milk and 20 g of table sugar per litre of water) (Lohmeyer & Pound 2012). The eggs obtained from the captured flies were either used directly in the ovicidal bioassays or transferred to plastic Solo cups containing a semi-synthetic diet used by Kumar *et al*. (2012) with slight modification (water based paste of wheat bran: 5 g wheat bran, 3 g milk powder, and 2 g sugar mixed with 10 mL of water) which was changed every second day until larvae reached the pupal stage. The larval stages and pupae obtained via this method were used in the bioassays.

### Ovicidal Activity

A stock solution of pomaces, seeds, and peels of two grape cultivars was prepared by dissolving them in boiling distilled water, and working solutions were prepared by further dilutions with distilled water. Two mL of the working solutions were pipetted into each Petri-dish together with 20 eggs and made up to 5 ml with distilled water to give final concentrations of the selected part ranging from 10 μg to 150 μg/mL.

The experiments were conducted in duplicate, and eggs in distilled water alone were used as controls. The commercial insecticide, Agita 10 WG, was used as a positive control. The eggs were incubated at room temperature (~24°C) for 24 h, after which a drop of Lugol’s iodine was added, to stop further hatching, and the numbers of unhatched eggs and L_1_ larvae were counted to determine the percentage inhibition of egg hatching by using the following equation:

% inhibition (ovicidal activity)=A-B/A×100

Where A represents the number of L_1_ larvae in the control incubation while B represents the number of L_1_ larvae in the experimental incubation.

### Larvicidal and Pupicidal Bioassays

The larval and pupicidal bioassays were evaluated by using dipping method (Sinthusiri & Soonwera 2013). Batches of 20 L_3_ larvae or pupae were immersed for 30 s in each aqueous extract containing different concentrations of the pomaces and their ingredients (seeds and peels). Three concentrations (10, 50, 100 mg/mL) of each tested extract along with control treatments free of the pomaces and their ingredients have been used. Each treatment was replicated twice while larvae and pupae in the control group were treated with distilled water only. The commercial insecticide, Agita 10 WG, was used as a positive control. After immersion, each batch of larvae or pupae was placed on filter paper to remove excess extract and then placed in Solo plastic cups and covered with facial tissue held in its place by rubber band and left for 2 h and then the larval mortality was recorded. The criterion for larval mortality was evaluated by softy touching each larva with a small paint brush and if they not responding were considered dead (Sinthusiri & Soonwera 2013) and percentage mortality was estimated using the following equation:

Percentage mortality (%)=[(A-B)/A]×100

Where A is the number of dead larvae in the untreated (control) Petri plates and B is the number of dead larvae in the treated Petri plates.

Concerning the pupicidal activity, the pupae were left for 7 days after dipping process and the activity of the pomaces and their ingredients (seeds and peels) was determined by estimating the percentage inhibition of adult emergence by using the following equation:

Pupicidal activity (%)=[(A-B)/A]×100

Where A is the number of emerged adult flies in the untreated (control) Petri plates and B is the number of emerged adults in the treated Petri plates.

### Development of L_1_ Larvae to Pupae and Adult Flies using Cow Manure

Cow manure was also used as a natural medium for the development of L_1_ larvae to L_3_, pupae and adult flies (Lohmeyer & Pound 2012). The manure was collected directly from the rectum (to avoid contamination with the soil) of the cows slaughtered at the local abattoir and placed in plastic bags and was frozen until needed for bioassays.

Two treatment placement locations were selected, top of the manure and bottom of manure. For top treatment, 100 g of manure were placed in the plastic Solo cups and then 5% or 10% of the powdered pomaces, seeds or peels were sprinkled on the top of the manure. For bottom treatment, the same amounts of powdered by-products were sprinkled on the bottom of the cup and then 100 g of manure were added to the cup. A similar quantity of untreated manure was used as a control treatment. Each treatment was replicated twice. After the addition of manure and the powdered pomaces, seeds, and peels to the cups, 20 L_1_ larvae were placed on top of the manure and the cups were covered with facial tissue held in place with a rubber band and left at 28°C for 7 days and observed for pupation. Pupae were collected by flotation (Lohmeyer & Pound 2012) and the numbers of normal and deformed pupae were recorded. For the adult emergence experiment, all pupae were left at 28°C and a photoperiod of 12:12 (light: dark) hours for another 7 days. The number of emerged adult flies was recorded for each sample and the percentage inhibition of development of L_1_ larvae to adults was calculated (Lohmeyer & Pound 2012).

### Statistical Analysis

Eggs, larvae, pupae and adult flies counts were expressed as mean ± standard error of the mean (SE). The significance of difference between the means was determined by one-way ANOVA using a computer software package (SPSS for Windows Release 20.0) and considered as significant when *p* ≤ 0*.*05.

## RESULTS

### Effect of Water Extracts on the Viability of Eggs

In the control wells 100% of the eggs hatched. Table 1 shows that the proportion of eggs hatching decreasing with increasing concentrations of powdered pomaces, seeds, and peels. It can be seen from Table 1 that the extracts from the seeds of Des-Alanez (DA) cultivar were significantly more effective (*p* < 0.05–0.001) at inhibiting the egg hatching than the extracts from the seeds of Shada cultivar. At 100 μg/mL, for example, the extracts from the seeds of DA cultivar were able to inhibit 61.4% of the eggs from hatching while the extracts of the Shada seeds inhibited 44.4% of the eggs from hatching. Similarly, the extracts from the pomaces of DA cultivar were significantly more effective (*p* < 0.05) than their counterparts from Shada cultivar (Table 1) while no significant differences were observed between the extracts from the peels of the two cultivars. At 150 μg/mL, the water extracts from the powdered pomaces, seeds, and peels were able to inhibit completely the hatching of the eggs (100% inhibition) when compared with the eggs in the control group (exposed to water only).

In the positive control group, the commercial insecticide Agita 10 WG was able to inhibit 67.8% of the eggs from hatching at a concentration of 31 μg/mL.

### Effect of Water Extracts on the Viability of L_3_ Larvae

The larvicidal activity of grape pomaces and their parts (peels and seeds) of two Iraqi grape cultivars using contact toxicity bioassay was found to be dependent mainly on concentrations used. The results of the present study (Table 2) showed that the crude water extracts of the pomaces, seeds, and peels of the two cultivars showed inhibitory effects against the viability of the L_3_ larvae as evidenced by their ability to kill the larvae and this ability increases significantly with increasing the concentration (*p* < 0.05). The results also revealed that the ability of extracts of the seeds of the two cultivars to kill the L_3_ larvae was significantly higher (*p* < 0.5-0.001) than the extracts of the pomaces and peels, especially at the higher concentrations.

### Effect of Water Extracts on the Development of L1 Larvae to Pupae and Adult Flies

The last series of the laboratory experiments are considered the most important experiments in this study because they simulate field conditions. The results of these experiments showed for the first time that the powdered pomaces, seeds, and peels of two grape cultivars have the ability to inhibit the development of up to 50% of the L_1_ larvae into pupae (Table 3) and up to 47.5% of L_1_ larvae into adult flies (Table 4) and this ability depends on the location of the products and the grape cultivar. The results also revealed that the addition of the powdered materials of both cultivars on top of the manure inhibited significantly more (*p* < 0.05) L_1_ larvae from reaching the pupa and adult stages in comparison with their counterparts which had been added at the bottom of the manure.

## DISCUSSION

Although the chemical control methods are very effective against flies, including the housefly, they have many disadvantages such as resistance to the chemical insecticides, toxic effects to humans and environment, and the high cost. Nowadays, the botanical insecticides have been given more attention to be alternative control methods against the harmful flies, including the housefly. The results of this study show that the powdered grape pomaces, seeds, peels, and their water extracts can disrupt the life cycle of the housefly, *Musca domestica*, by preventing their eggs from hatching and by preventing the development of L_I_ larvae to L_3_, pupae and adult stages. As far as we know, this is the first demonstration that the grape pomaces and/or their ingredients can disrupt the life cycle of the housefly.

The boiling water has been used in this study because our previous studies have shown that boiling water was a good solvent for extracting phenolic compounds and even more effective than some organic solvents (Molan & Mahdy, 2016; Molan *et al.* 2017). In addition, the most common extraction method in herbal medicine is to boil the herb in hot water and it has been suggested that water is one of what they are called ‘green’ solvents (Hailes 2007; Simon & Lee 2012).

Although the mechanisms by which the water extracts of the powdered pomaces, seeds and peels of grape cultivars inactivate the eggs are not identified, they may inactivate enzymes responsible for the hatching process. Polyphenolic compounds, especially condensed tannins have been shown to inhibit endogenous enzyme activities in rat intestine (Oh & Hoff 1986, Horigome *et al*. 1988) and they were potent inhibitors of cyclic AMP-dependent protein kinase in rat liver (Wang *et al*. 1996). The hatching of nematode eggs is initiated by environmental stimuli that lead to the release of so called ‘hatching enzymes’ (Sommerville & Rogers 1987) which include proteases, lipases, chitinases, 1-glycosidases and leucine aminopeptidases. The inhibition of some of these enzymes has been shown to reduce the rate of hatching or even to stop the process completely (Rogers & Brooks 1977; Arnold *et al*. 1993). The larvae of the housefly are morphologically similar to the nematodes infecting humans, especially *Enterobius vermicularis* so they have been used as experimental models for studying the anthelmintic activity of some anthelmintic drugs (Murugamani *et al*. 2012). Accordingly, the larvicidal activity of the aqueous extracts prepared from grape pomaces and their components (peels and seeds) against the larvae of the housefly demonstrated in the present study may be considered as an indication for the anthelmintic activity.

We believe that when the powdered pomaces, seeds, peels and/or their water soluble extracts are added to the media containing eggs, larvae, pupae, and adults of the housefly, the phenolic compounds present in powdered samples could either have internal effect when they have been swallowed by the larvae or external effect when they interact with the protein surface of the eggs, larvae, and pupae. Previously, Molan, Alexander *et al*. (2000), Molan, Duncan *et al.* (2000), Molan, Hoskin *et al.* (2000) Molan, Waghorn, *et al.* (2000), Molan *et al.* (2001; 2002), Molan and Farag (2010) and Molan (2014) studied the effect of condensed tannins from different sources on the viability of eggs and larvae of sheep and deer nematodes and found that the tannins affect them both internally and externally. When the viability of the trapped larvae, which could not pass through the nylon mesh sieves of the larval migration inhibition (LMI) bioassay, was checked it was found that 83% to 93% of the larvae were alive although their movements were sluggish, suggesting partial paralysis (Molan, Waghorn *et al*. 2000). As long as tannins can paralyse the body musculature of the nematodes, it is logical to predict that they may also paralyse the pharyngeal muscles and prevent the larvae and adult stages of the housefly from feeding.

In addition, the phenolic compounds found in the pomaces and their parts may bind with all components of the medium and make them unpalatable. This may explain the inhibitory effects of the powdered pomaces and their parts when they were added on top of the cow manure as the results showed that the powdered pomaces, seeds and peels of both cultivars showed significantly higher (*p* < 0.05) inhibitory effect against the L_1_ larvae when added on top of the manure in comparison to the control treatment which may indicate that the phenolic compounds present in the powdered by-products can mix and bind with the parts of the cow manure and affect the growth and development of the larvae into pupae and adult flies. This finding may indicate that the application of the powdered pomaces and/or their parts on the outer surface of the pens, barns and stables could be a good manure management and prevent the breeding of the flies. The hypothesis is that following sprinkling of ground pomaces on the floor (bedding material); the movement of the birds/farm animals will help and ensure the mixing of the pomace with the feces and consequently exposing the eggs and larval stages of flies and other pathogens to the negative effect of the pomaces.

Moreover, the swallowed phenolic compounds may affect negatively the absorption of nutrients via affecting the size and length of the intestinal villi (Nyamambi *et al*. 2007).

The insecticidal activity of the powdered pomaces, seeds, peels and their water soluble extracts against the eggs, larvae, pupae, and adults of the housefly differed significantly, with the seeds showed the highest activity followed by the pomaces and then peels. These differences in activity may be attributed to differences in the structure and molecular weight of phenolic compounds founds in the powdered samples (Waghorn *et al*. 2006).

All the concentrations and location placement combinations of the powdered pomaces, seeds and peels evaluated in this study reduced adult housefly emergence. The presence of deformed pupae indicated that the powdered grape pomaces, seeds and peels may have insect growth regulator (IGR) effects on the development of the housefly. Lohmeyer and Pound (2012) studied the insecticidal activity of the novaluron insecticide against three flies, including the housefly and they considered the presence of deformed pupae as an indication for the IGR effects on the development of the selected flies, resulting in larval mortality before they reached pupation or deformed pupae from which adults never emerged.

## CONCLUSION

The results of this study suggest that the powdered grape pomaces, seeds, and peels and their water soluble extracts may be able to break the life cycle of the housefly and thus could help to decrease the need for insecticides as the principal method of control. Moreover, botanical pesticides offer an advantage over synthetic insecticides as they can be much less toxic, less prone to the development of resistance and more easily degraded.

## Figures and Tables

**Table 1:** Percentage inhibition of housefly egg hatching after exposure for 24 h to aqueous extracts containing different concentrations of the powdered pomaces, seeds and peels of two Iraqi grape cultivars (Des-Alanez and Shada). The values are the means of two independent experiments ± standard error. The commercial insecticide, Agita 10 WG, has been used as a positive control.

Concentration (μg/mL) of pomaces, peels and seeds	% inhibition/ grape cultivar	

	Des-Alanez	Shada
Peels		
50	4.4 ± 1.5^a^	3.1 ± 1.1^a^
75	7.9 ± 0.7 ^a^	6.5 ± 1.5 ^a^
100	26.4 ± 2.2^a^	23.3 ± 3.3^a^
150	100.0 ± 0.0 ^a^	100.0 ± 0.0 ^a^
Seeds		
50	11.6 ± 1.1^a^	9.3 ± 0.7^b^
75	15.4 ± 0.2^a^	12.2 ± 2.2^b^
100	61.4 ± 1.4^a^	44.4 ± 5.6^b^
150	100.0 ± 0.0 ^a^	100.0 ± 0.0 ^a^
Pomace		
50	7.7 ± 0.95^a^	3.0 ± 0.6^b^
75	12.4 ± 2.4^a^	8.0 ± 0.9^b^
100	43.1 ± 1.4^a^	39.6 ± 3.2^b^
150	100.0 ± 0.0 ^a^	100.0 ± 0.0 ^a^
Negative control group	0.0 ± 0.0	0.0 ± 0.0
Positive control group (Agita 10 WG)		
31 μg/mL	67.1 ± 0.42	67.1 ± 0.42
50 μg/mL	100.0 ± 0.0	100.0 ± 0.0

The values in the same row with different superscript letters differ significantly (*p* ≤ 0.05).

**Table 2:** Percentage mortality of L_3_ 2 h after dipping in aqueous extracts of powdered pomaces, seeds and peels of two grape cultivars. The values were the means of two independent experiments ± standard error. The commercial insecticide, Agita 10 WG, was used as a positive control.

% mortality of L_3_ larvae (2 h after dipping in the extracts)		

Part/ con. (mg/mL)	Des Al-Anez	Shada
Peels		
10	15 ± 5^a^	10 ± 0^a^
50	15 ± 5^a^	15 ± 5^a^
100	20 ± 0 ^a^	15 ± 5 ^a^
Seeds		
10	15 ± 5 ^a^	10 ± 0 ^a^
50	30 ± 0 ^a^	20 ± 0 ^b^
100	50 ± 5 ^a^	30 ± 0 ^b^
Pomace		
10	15 ± 5^a^	10 ± 0^b^
50	25 ± 5^a^	20 ± 0^a^
100	25 ± 5 ^a^	25 ± 5 ^a^
Negative control (water)	0	0
Positive control (Agita, 31μg/mL)	45 ± 5 ^a^	45 ± 5 ^a^

The values in the same row with different superscript letters differ significantly (*p* ≤ 0.05).

**Table 3:** The percentage inhibition of the development of housefly L_1_ larvae into pupae after the addition of powdered pomaces, seeds and peels of two Iraqi grape cultivars on top and bottom of the cow manure. The values are the means ± standard errors of two separate experiments and the number of larvae was 20 in each treatment and each experiment.

% inhibition in development of L_1_ larvae into pupae				

Part/Con.	Des Al-Anez		Shada	
	
	Top layer	Bottom layer	Top layer	Bottom layer
Peels				
5%	22.5 ± 1.4^a^	17.5 ± 1.4 ^b^	19.0 ± 5.1 ^a, b^	20.0 ± 5.6 ^a, b^
10%	32.5 ± 1.4 ^a^	27.5 ± 1.4 ^b^	22.5 ± 1.4 ^c^	32.5 ± 1.9 ^a^
Seeds				
5%	32.5 ± 1.4 ^a^	20.0 ± 0.0 ^b^	27.0 ± 3.9 ^c^	23.0 ± 7.3 ^b, c^
10%	50.0 ± 2.8 ^a^	50.0 ± 0.0 ^a^	33.0 ± 1.1 ^b^	34.0 ± 0.6 ^b^
Pomace				
5%	22.5 ± 1.4 ^a^	20.0 ± 2.8 ^a^	22.5 ± 9.9 ^a^	26.0 ± 9.0 ^a^
10%	32.5 ± 1.4 ^a^	27.5 ± 1.4 ^b^	28.5 ± 4.8 ^a, b^	29.5 ± 5.4 ^a, b^

The values in the same row with different superscript letters differ significantly (*p* ≤ 0.05).

**Table 4:** The percentage inhibition of the development of housefly L_1_ larvae into adult flies after the addition of powdered pomaces, seeds and peels of two Iraqi grape cultivars on top and bottom of the cow manure. The values are the means ± standard errors of two separate experiments and the number of larvae was 20 in each treatment and each experiment.

% inhibition in development of L1 larvae into adult				

Part/Con.	Des Al-Anez		Shada	
	
	Top layer	Bottom layer	Top layer	Bottom layer
Peels				
5%	12.5 ± 1.4 ^a^	7.5 ± 1.4 ^b^	5.0 ± 2.8 ^b^	5.0 ± 2.8 ^b^
10%	20 ± 0 ^a^	17.5 ± 1.4 ^b^	12.5 ± 1.4 ^c^	10.0 ± 2.8 ^c^
Seeds				
5%	22.5 ± 1.4 ^a^	15.0 ± 2.8 ^b^	7.5 ± 1.4 ^c^	2.5 ± 1.4 ^d^
10%	47.5 ± 1.4 ^a^	32.5 ± 1.4 ^b^	27.5 ± 1.4 ^c^	17.5 ± 1.4 ^d^
Pomace				
5%	12.5 ± 1.4 ^a^	7.5 ± 1.4 ^b^	2.5 ± 1.4 ^c^	2.5 ± 1.4 ^c^
10%	30.0 ± 0.0 ^a^	20.0 ± 0.0 ^b^	17.5 ± 1.4 ^c^	12.5 ± 1.4 ^d^

The values in the same row with different superscript letters differ significantly (*p* ≤ 0.05).

## References

[b1-tlsr-29-2-89] Arnold K, Brydon LJ, Chappel LH, Googay GW (1993). Chitinolytic activities in Heligmosomoides polygyrus and their role in egg hatching. Molecular and Biochemical Parasitology.

[b2-tlsr-29-2-89] Bustamante MA, Moral R, Paredes C, Pérez-Espinosa A, Moreno-Caselles J, Pérez-Murcia MD (2008). Agrochemical characterisation of the solid by-products and residues from the winery and distillery industry. Waste Management.

[b3-tlsr-29-2-89] Curko NC, Ganic KK, Gracin L, Dapic M, Jourdes M, Teissedre PL (2014). Characterization of seed and skin polyphenolic extracts of two red grape cultivars grown in Croatia and their sensory perception in a wine model medium. Food Chemistry.

[b4-tlsr-29-2-89] Devesa-Rey R, Vecino X, Varela-Alende JL, Barral MT, Cruz JM, Moldes AB (2011). Valorization of winery waste vs. the costs of not recycling. Waste Management.

[b5-tlsr-29-2-89] Farooq M, Freed S (2016). Infectivity of housefly, *Musca domestica* (Diptera: Muscidae) to different entomopathogenic fungi. Brazilian Journal of Microbiology.

[b6-tlsr-29-2-89] Food and Agriculture Organization (FAO) (2009). The state of food and agriculture.

[b7-tlsr-29-2-89] Hailes HC (2007). Reaction solvent selection: the potential of water as a solvent for organic transformations. Organic Process Research and Development.

[b8-tlsr-29-2-89] Horigome T, Kumar R, Okamoto K (1988). Effects of condensed tannins prepared from leaves of fodder plants on digestive enzymes *in vitro* and in the intestine of rats. British Journal of Nutrition.

[b9-tlsr-29-2-89] Kammerer D, Claus A, Carle R, Schieber A (2004). Polyphenol screening of pomace from red and white grape varieties (*Vitis vinifera* L.) by HPLC–DAD–MS/MS. Journal of Agricultural and Food Chemistry.

[b10-tlsr-29-2-89] Khan HAA, Shad SA, Akram W (2013). Resistance to new chemical insecticides in the house fly, *Musca domestica* L., from dairies in Punjab, Pakistan. Parasitology Research.

[b11-tlsr-29-2-89] Kumar P, Mishra S, Malik A, Satya S (2012). Compositional analysis and insecticidal activity of *Eucalyptus globulus* (family: Myrtaceae) essential oil against housefly (*Musca domestica*). Acta Tropica.

[b12-tlsr-29-2-89] Lee-Jones D (2012). New Zealand 2012 wine and vineyard report.

[b13-tlsr-29-2-89] Lohmeyer KH, Pound JM (2012). Laboratory evaluation of novaluron as a development site treatment for controlling larval horn flies, house flies, and stable flies (Diptera: Muscidae). Journal of Medical Entomology.

[b14-tlsr-29-2-89] Lurton L (2003). Grape polyphenols: New powerful health ingredients. Innov Food Technology.

[b15-tlsr-29-2-89] Malik A, Singh N, Satya S (2007). *Musca domestica* (housefly): a challenging pest and the control strategies. Journal of Environmental Science and Health: Part B.

[b16-tlsr-29-2-89] Mansour SA, Ibrahim RM, El-Gengaihi SE (2014). Insecticidal activity of chicory (*Cichorium intybus* L.) extracts against two dipterous insect-disease vectors: Mosquito and housefly. Industrial Crops and Products.

[b17-tlsr-29-2-89] Molan AL, Farag AM (2010). The effects of condensed tannins extracted from different plant species on egg hatching and larval development of *Teladorsagia circumcinicta* (Nematoda: Trichostrongylidae). Folia Parasitologica.

[b18-tlsr-29-2-89] Molan AL, Mahdy AS (2016). Total phenolics, antioxidant activity and anti-diabetic capacities of selected Iraqi medicinal plants. American Journal of Life Science Researches.

[b19-tlsr-29-2-89] Molan AL, Alexander R, Brookes IM, McNabb WC (2000). Effects of sulla condensed tannins on the viability of three sheep gastrointetinal nematodes *in vitro*. Proceedings of the New Zealand Society of Animal Production.

[b20-tlsr-29-2-89] Molan AL, Atwood GT, Min BR, McNabb WC (2001). The effect of condensed tannins extracted from two lotus species on the growth of proteolytic rumen bacteria *in vitro* and their possible mode of action. Canadian Journal of Microbiology.

[b21-tlsr-29-2-89] Molan AL, Duncan A, Barry TN, McNabb WC (2000). Effects of condensed tannins and sesquiterpene lactones extracted from chicory on the viability of deer lungworm larvae. Proceedings of the New Zealand Society of Animal Production.

[b22-tlsr-29-2-89] Molan AL, Hoskin SO, Barry TN, McNabb WC (2000). The effect of condensed tannins extracted from four forages on deer lungworm and gastrointestinal nematode larval viability. Veterinary Record.

[b23-tlsr-29-2-89] Molan AL, Waghorn GC, McNabb WC (2002). The impact of condensed tannins on egg hatching and larval development of *Trichostrongylus colubriformis in vitro*. Veterinary Record.

[b24-tlsr-29-2-89] Molan AL, Waghorn GC, Min BR, McNabb WC (2000). The effect of condensed tannins from seven herbages on *Trichostrongylus colubriformis* larval migration *in vitro*. Folia Parasitologica.

[b25-tlsr-29-2-89] Molan AL, Yousif AA, Al-Bayati NY (2017). Total phenolic contents and antiradical activities of pomaces and their ingredients of two Iraqi date cultivars. World Journal of Pharmacy and Pharmaceutical Sciences.

[b26-tlsr-29-2-89] Molan AL (2014). Effect of purified condensed tannins from pine bark on larval motility, egg hatching and larval development of *Teladorsagia circumcincta* and *Trichostrongylus colubriformis* (Nematoda: Trichostrongylidae). Folia Parasitologica.

[b27-tlsr-29-2-89] Morre DM, Morre DJ (2005). Anticancer activity of grape and grape skin extracts alone and combined with green tea infusions. Cancer Letters.

[b28-tlsr-29-2-89] Murugamani V, Raju L, Raj VBA, Kataki MS, Sankar GG (2012). The new method development for evaluation of anthelmintic activity by housefly worms and compared with conventional earth worm method. International Scholarly Research Network.

[b29-tlsr-29-2-89] Nyamambi B, Ndlovu LR, Naik YS, Kock ND (2007). Intestinal growth and function of broiler chicks fed sorghum based diets differing in condensed tannin levels. South African Journal of Animal Science.

[b30-tlsr-29-2-89] Oh HI, Hoff JE (1986). Effect of condensed grape tannins on the *in vitro* activity of digestive proteases and activation of their zymogens. Journal of Food Science.

[b31-tlsr-29-2-89] Ozkan G, Sagdic O, Gokturk-Baydar N, Kurumahmutoglu Z (2004). Antibacterial activities and total phenolic contents of grape pomace extracts. Journal of the Science of Food and Agriculture.

[b32-tlsr-29-2-89] Palacios SM, Bertoni A, Rossi Y, Santander R, Urzua A (2009). A insecticidal activity of essential oils from native medicinal plants of Central Argentina against the housefly, *Musca domestica* (L.). Parasitology Research.

[b33-tlsr-29-2-89] Rockenbach II, Rodrigues E, Gonzaga LV, Caliari V, Genovese MI, Fett R (2011). Phenolic compounds content and antioxidant activity in pomace from selected red grapes (*Vitis vinifera* L. and *Vitis labrusca* L.) widely produced in Brazil. Food Chemistry.

[b34-tlsr-29-2-89] Rogers WP, Brooks F (1977). The mechanism of hatching of eggs of *Haemonchus contortus*. International Journal for Parasitology.

[b35-tlsr-29-2-89] Scott JG, Leichter CA, Rinkevihe FD, Harris SA (2013). Insecticide resistance in house flies from the United States: Resistance levels and frequency of pyrethroid resistance alleles. Pesticide Biochemistry and Physiology.

[b36-tlsr-29-2-89] Simon MO, Lee CJ (2012). Green chemistry oriented organic synthesis in water. Chemical Society Reviews.

[b37-tlsr-29-2-89] Sinthusiri J, Soonwera M (2013). Efficacy of herbal essential oils as insecticides against the house fly, *Musca domestica* L. Southeast Asian Journal of Tropical Medicine and Public Health.

[b38-tlsr-29-2-89] Sommerville RI, Rogers WP (1987). The nature and action of host signals. Advanced Parasitology.

[b39-tlsr-29-2-89] Waghorn TS, Molan AL, Deighton M, Alexander RA, Leathwick DM, McNabb WC, Meagher LP (2006). *In vivo* anthelmintic activity of *Dorycnium rectum* and grape seed extract against *Ostertagia (Teladorsagia) circumcincta* and *Trichostrongylys colubriformis* in sheep. New Zealand Veterinary Journal.

[b40-tlsr-29-2-89] Wang BH, Foo LY, Polya GM (1996). Differential inhibition of eukaryote protein kinases by condensed tannins. Phytochemistry.

[b41-tlsr-29-2-89] Zhu F, Du B, Zheng L, Li J (2015). Advance on the bioactivity and potential applications of dietary fibre from grape pomace. Food Chemistry.

